# Temperature-Compensated Solution Concentration Measurements Using Photonic Crystal Fiber-Tip Sensors

**DOI:** 10.3390/s23187703

**Published:** 2023-09-06

**Authors:** Mildred S. Cano-Velázquez, Arthur L. Hendriks, Luca Picelli, Rene P. J. van Veldhoven, Andrea Fiore

**Affiliations:** 1 Department of Applied Physics and Science Education, Eindhoven University of Technology, 5600 MB Eindhoven, The Netherlands; a.l.hendriks@tue.nl (A.L.H.); l.picelli@tue.nl (L.P.); p.j.veldhoven@tue.nl (R.P.J.v.V.); a.fiore@tue.nl (A.F.); 2 Eindhoven Hendrik Casimir Institute, Eindhoven University of Technology, 5600 MB Eindhoven, The Netherlands

**Keywords:** optical fiber sensors, photonic crystals, chemical sensors, in-line measurements

## Abstract

We demonstrate fiber optic sensors with temperature compensation for the accurate measurement of ethanol concentration in aqueous solutions. The device consists of two photonic crystal (PhC) fiber-tip sensors: one measures the ethanol concentration via refractive index (RI) changes and the other one is isolated from the liquid for the independent measurement of temperature. The probes utilize an optimized PhC design providing a Lorentzian-like, polarization-independent response, enabling a very low imprecision (pm-level) in the wavelength determination. By combining the information from the two probes, it is possible to compensate for the effect that the temperature has on the concentration measurement, obtaining more accurate estimations of the ethanol concentration in a broad range of temperatures. We demonstrate the simultaneous and single-point measurements of temperature and ethanol concentration in water, with sensitivities of 19 pm/°C and ∼53 pm/%, in the ranges of 25 °C to 55 °C and 0 to 50% (at 25 °C), respectively. Moreover, a maximum error of 1.1% in the concentration measurement, with a standard deviation of ≤0.8%, was obtained in the entire temperature range after compensating for the effect of temperature. A limit of detection as low as 0.08% was demonstrated for the concentration measurement in temperature-stable conditions.

## 1. Introduction

Fiber optic sensors (FOS) allow passive, real-time, and remote sensing over large distances, do not suffer from electromagnetic interference, and can be bio-compatible and inert to most chemical hazards and elevated temperatures. Additionally, FOSs have a small size, are lightweight, and, differently from bulk optical sensing, pose fewer requirements in terms of alignment and coupling [1]. Due to these advantages, optical fiber sensors have been used to measure diverse parameters, among which the refractive index (RI) of the external medium has attracted much interest. This is mainly because the refractive index of a solution is useful in identifying and determining the concentration of substances, which is relevant for a wide range of applications, such as chemical analysis, food testing, environmental monitoring, biological detection, and medical diagnosis [2]. A large variety of optical fiber sensors has been proposed as an alternative to the standard methods for refractive index sensing and concentration measurements, such as the Abbe refractometers [3]. In view of their miniaturized size and versatility, fiber optic sensors represent an attractive solution for in-line concentration measurements.

As a study case, we consider here the case of ethanol concentration measurement in aqueous solutions. Since ethanol is extensively used in several sectors including the medical, chemical, pharmaceutical, beverage, and fuel industries, there is a growing interest in the proper monitoring of its concentration during numerous industrial processes [4,5]. In particular, for the beverage and fuel industries, the adulteration of ethanol with water is a common issue that can be addressed by a proper determination of the ethanol concentration during the whole process [6]. In general, the precise measurement of the ethanol concentration in an aqueous solution provides important information regarding the quality of the process and the final product [5,7]. An in-line monitoring of ethanol concentration has obvious advantages in terms of cost and real-time data availability over offline solutions [4,6]. Multiple FOS-based approaches for the measurement of ethanol concentrations have been proposed in the last few years, such as evanescent wave-based sensors [8,9,10], fiber Bragg and long period gratings [6,11,12,13], tapered FOS [7], plasmonic sensors [14,15] and special fiber-based sensors [16]. Regardless of the advantages and disadvantages of each sensor, in general, temperature cross-sensitivity represents a significant issue [4]. Since FOSs generally respond to temperature changes, different approaches have been proposed in order to mitigate this problem [11,12,17,18,19]. However, these proposed devices have additional drawbacks including complex fabrication methods, use of delicate structures or specialized fiber optics, and difficult signal demodulation [4].

In this paper, we present a simple sensing configuration for the determination of ethanol concentration with temperature compensation. The proposed sensor probe is based on photonic crystal (PhC) fiber-tip sensors. Each sensor is assembled with a simple and controllable method, where a 2D photonic crystal is fabricated on a wafer by standard semiconductor fabrication methods, and then it is mechanically transferred to the fiber end-face [20]. This transfer method only requires a movable stage and a microscope camera, and different to similar techniques, it does not need adhesives or additional materials to maintain the PhC attached to the fiber. These devices have shown a strong spectral response in reflection, and their applicability in temperature and refractive index sensing has been demonstrated [20]. While one of these sensors can be used to measure the solution’s refractive index, which is correlated to the ethanol concentration in the liquid mixture, its sensitivity to temperature needs to be compensated. By placing a second sensor in close proximity to the concentration sensor and isolating the fiber tip from the external medium, the temperature can be retrieved simultaneously to compensate for the temperature effect on the concentration measurement of the first sensor. This sensor probe design and the temperature fluctuation compensation provide a reliable measure of ethanol concentrations over a broad temperature range (25 °C to 55 °C), reducing the maximum absolute error in the measured concentration, from 33.7% to 1.1%.

## 2. Materials and Methods

### 2.1. PhC Fiber-Tip Sensor Fabrication

**Figure 1:**
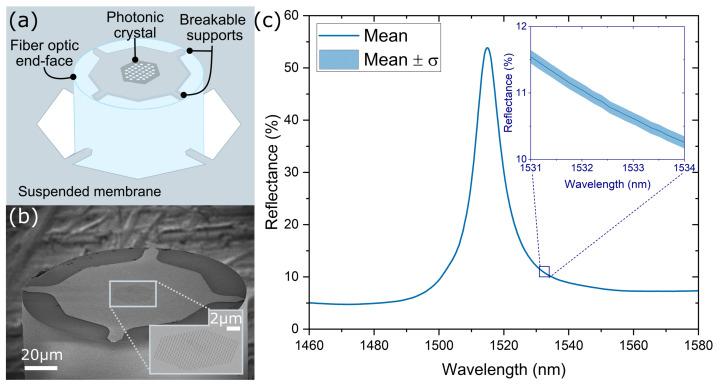
(**a**) Schematic representation of the transfer method of the PhC to the fiber end-face. (**b**) SEM image of the PhC fiber-tip sensor, inset: zoom-in on the PhC. (**c**) Average (solid line) and standard deviation (σ) (shaded region) of the reflection spectra obtained from ten different polarization states.

The photonic crystals are fabricated using established semiconductor fabrication processes. Once the PhC structure is fabricated, it is mechanically transferred to the facet of the optical fiber. This transfer mechanism is facilitated by establishing a suspended membrane surrounding the sensor structure and aligning it with the fiber via a predefined hole within the substrate. By using an optimized support structure, adhesion forces ensure the contact between the membrane and the fiber facet as the fiber is gradually introduced onto the structure, leading to the eventual breakage of the supports (see Figure 1a).

For this work, PhC structures with a hexagonal lattice were patterned on a 250 nm thick InP membrane, which is separated from a InP substrate by a 300 nm thick sacrificial InGaAs layer, following the fabrication methods described in [20]. Briefly, the photonic crystal is patterned on the InP membrane by electron beam lithography (EBL) followed by inductively coupled plasma reactive ion etching (ICP-RIE), using a SiN layer as a hard mask. For the openings on the back of the wafer, SiN layers are deposited on both sides of the sample. The SiN layer on the backside is patterned with rectangles that are aligned with the PhC structures, and the top layer acts to protect the InP membrane during the subsequent steps. Finally, the substrate, sacrificial layer, and SiN layers are selectively etched, resulting in a suspended InP membrane with a PhC structure with back access for the fiber.

For the transfer of the optical structures to the fiber tip, a wafer is placed on a holder, allowing access of the fiber to the PhC structures from the bottom side and allowing the visualization of the PhC structures from the top side with a microscope camera. A cleaved single-mode fiber is placed vertically below the PhC structure on a movable stage; a white light source is coupled to the fiber to assist the localization of the fiber core with the camera. As the fiber is elevated, the core is aligned with the center of the PhC. During this process, the distance and optical response of the PhC structure can be monitored by measuring continuously the reflection spectra with an optical interrogator (Micron Optics Hyperion si255, Luna, Roanoke, VA, USA), based on a swept-wavelength laser source with an output power of 0.25 mW [21]. Once the fiber core and optical structure are aligned, the fiber facet is brought into contact with the suspended structure, causing the four lateral supports to break. Figure 1b is an SEM image of the final PhC fiber-tip sensor. After the transfer and first immersion in a liquid, the spectrum is very stable and reproducible, as we reported previously [20].

The reflection spectrum of the fiber-tip PhC was measured with the same optical interrogator and normalized using a fiber optic retroreflector (P5-SMF28ER-P01-1, Thorlabs, Newton, NJ, USA), to provide the reflectance. As can be seen in Figure 1c, the response of the fabricated PhCs has a shape close to a Lorentzian ($\lambda_{0}=1515.1$ nm, ΔR=49.4%, full-width half-maximum FWHM=10.0 nm, quality factor Q=151) and is polarization-independent. We note that photonic crystals operated at the Γ point tend to show a Fano-like reflection spectrum [20] due to the interference between the reflection from the planar multilayer and the backscattering from the PhC. To approximate the PhC response to a Lorentzian lineshape, the thickness of the InP layer is chosen so that the reflectance from the planar multilayer structure is minimized. Additionally, the PhC with a hexagonal lattice, different from the ones with band-folded rectangular lattice [20], presents the advantage of having a polarization-degenerate mode which produces a response that does not depend on the polarization state of the incident light [22]. To test this, the reflected spectrum is measured for ten different polarization states, selected arbitrarily using a manual fiber polarization controller (FPC562, Thorlabs). The mean and standard deviation of the measured reflectance spectrum are shown in Figure 1c. These results display a maximum standard deviation (σ) of 0.12%. In the reflection spectra obtained during the experiments, we did not detect any influence from unintended reflections originating from the surroundings of the sensing probe being directed back into the fiber.

### 2.2. Sensor Probe Design and Spectral Response

**Figure 2:**
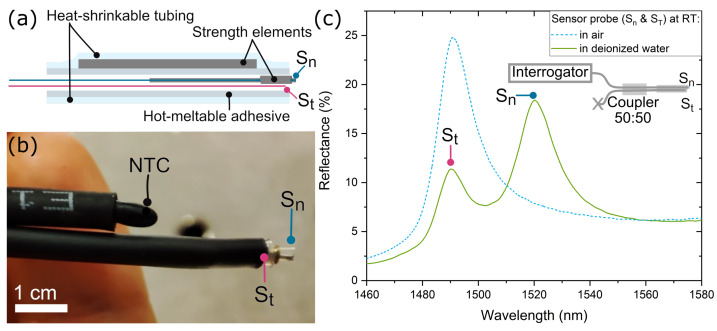
(**a**) Schematic of the sensing probe design. (**b**) Photo of the sensor probe, including a PhC refractive index ($S_{n}$) and a temperature ($S_{t}$) sensors. An NTC thermistor is bundled to the sensing probe to measure the reference temperature. (**c**) Experimental measurement of the sensor probe response at RT in air and after being immersed in deionized water. The inset shows a schematic of the sensor probe made of the two $S_{n}$ and $S_{t}$ sensors and the interrogation approach.

For the sensor probe, two PhC fiber-tip sensors are assembled in the same device: the refractive index sensor ($S_{n}$), which will be exposed to the solution to analyze, and the temperature sensor ($S_{t}$), which must remain isolated from the liquid (see Figure 2). As a means to protect $S_{n}$ and to better visualize its position, a tailor-made metallic strength element was designed to surround this sensor. Once $S_{n}$ is inserted and glued to the metal structure, both sensors are placed in a fiber optic splice protection sleeve. When this sleeve is heated, the inner tubing melts and the external tubing shrinks around the fiber optics. With the purpose of encapsulating $S_{t}$ with the melted polymer, its facet is placed 5 mm before the end of the sleeve using a microscope and a movable stage. In contrast, $S_{n}$, with its strength element, is placed outside the heat-shrinkable tube. When the whole device is heated, the melted polymer maintains all the elements in place. Figure 2a shows a schematic representation of the elements that compose the sensing probe, and Figure 2b is an image of the final sensing probe. The size comparison with a commercial NTC (Negative Temperature Coefficient) thermometer (TSP-TH, Thorlabs) can be appreciated in the same picture. This thermometer is used as a reference temperature sensor for some of the experiments explained in the following sections.

In order to read out both sensors using a single interrogation channel, reducing the cost of the interrogating system, we propose the use of an optical coupler to combine the sensor outputs into a single spectrum. By tailoring the design of the PhCs, it is possible to tune the wavelength resonance of the fiber-tip sensors. This allows us to spectrally separate the two resonances, which facilitates the proper interrogation of both sensors. We aimed to match the wavelength resonance of $S_{n}$ and $S_{t}$ when placed within the sensor probe in air at ambient conditions. In this way, when the sensor probe is immersed in the liquid, the resonance of $S_{n}$ shifts away from $S_{t}$. Since $S_{t}$ is covered by a polymer, the spectral shift due to the change in the surrounding refractive index was measured previously and taken into account for the final design of the corresponding PhC. The parameters used for the PhCs were a lattice constant of 785 nm and 755 nm, and a hole radius of 98 nm and 105 nm, for $S_{n}$ and $S_{t}$, respectively. Figure 2c shows the final spectral response of the sensor probe, consisting of a 50/50 coupler (TW1550R5A1, Thorlabs) with $S_{n}$ and $S_{t}$ at the two end ports (inset Figure 2c), at room temperature (RT) when it is in air and when it is immersed in deionized water. It can be seen that both resonances, from $S_{t}$ and $S_{n}$, are clearly visible and measurable when the probe is in liquid. Notice that, since the coupler splits the incident and back-reflected light, the total reflectance for this configuration is lower than when one sensor is connected directly to the interrogator. As the liquid temperature is not controlled, a slight difference in the wavelength of $S_{t}$ for when the sensor probe is in air and in deionized water is visible.

## 3. Results and Discussion

### 3.1. Sensor Probe Characterization

Since the spectral response of the sensor probe is concentration- and temperature-dependent, the sensitivity to both parameters must be determined. The temperature sensitivity of the sensor $S_{t}$ is measured by placing the sensor probe in water at different temperatures. For the behavior of $S_{n}$, which depends on the concentration and the temperature, the sensor spectral response was evaluated for different water-ethanol solutions at different temperatures. Ethanol aqueous solutions with different volume fractions $\left({\%}_{V/V}\right)$ have different concentrations, and consequently, different refractive indices. Therefore, measurements of samples ranging from 0 to 75 $\left({\%}_{V/V}\right)$ are performed. For that, a centrifuge tube is inserted in the thermal block of a temperature-controlled mixer (Thermomixer Comfort, Eppendorf, Germany.) Then, the instrument is set to a constant temperature of 25 °C and a mixing (shaking) frequency of 300 rpm for tempering the tube. Then, the sensor probe is placed inside the tube and water is added. Since the water is at a different temperature than the tube, it is necessary to wait until the liquid and the container reach thermal equilibrium. After tempering the liquid, the sensors $S_{n}$ and $S_{t}$ show a stable response, and the wavelength resonance in water is measured for 10 min. Then, ethanol is added every 10 min to achieve the desired volume fractions (0, 25, 50, 75 $\left({\%}_{V/V}\right)$). The resonant wavelength of the sensor $S_{n}$ and the temperature reference is measured every second during the whole experiment. Subsequently, the same experiment is repeated for different temperatures (i.e., 35, 45, and 55 °C). This temperature range covers a wide range of practical operating conditions, including ambient temperatures and typical process temperatures in various industries [5]. It is important to notice that the upper limit of this range is constrained by the temperature accuracy of the thermo-controlled mixer, which experiences a notable decrease in temperature accuracy beyond 45 °C [23]. This setup limitation should not be considered as indicative of the sensor performance, which has previously demonstrated its capability in accurately sensing temperatures up to approximately 80 °C [20]. For both series of experiments, an NTC is placed close to the sensing probe in order to have a temperature reference. A virtual instrument was designed for the spectra acquisition, the determination of the resonance wavelength ($\lambda_{0}$), and the display and storage of this information. For the determination of each resonance wavelength, the virtual instrument localizes the maximum of each peak from the spectra acquired with the optical interrogator, selects a 40 nm window centered at the localized maximum, and fits this region with a Lorentzian function.

**Figure 3:**
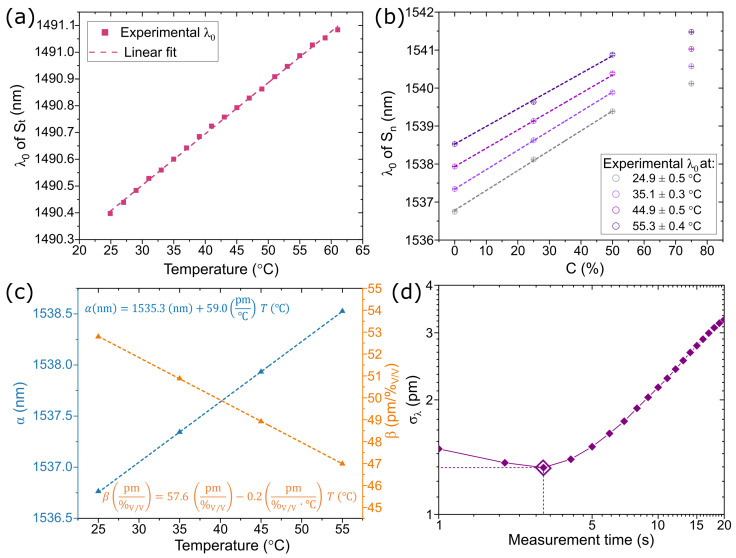
(**a**) Resonant wavelength for the sensor $S_{t}$ for when the sensing probe is immersed in water while changing the temperature. (**b**) $S_{n}$ resonant wavelength dependence on volume fraction for ethanol-water mixtures for different temperatures. (**c**) Intercept (α) and slope (β) values obtained from the linear fit of the wavelength dependence on concentration for different temperatures. (**d**) Allan deviation as a function of measurement time for the resonant wavelength of the sensor $S_{n}S$ acquired during 8 min in water at 25 °C.

Figure 3a shows the resonant wavelength of the encapsulated sensor ($S_{t}$) when the probe is immersed in deionized water at different temperatures. For this parameter, the achieved sensitivity of $S_{t}$ is $\kappa_{T}^{S_{t}}=19.1$ pm/°C. Similarly, Figure 3b presents the variation of the resonant wavelength of the concentration sensor $S_{n}$ with volume fraction for the analyzed ethanol-water mixtures at different temperatures. The resonant wavelengths presented in Figure 3b are the 8 min averaged values, and the error bars represent the standard deviation over the same time interval. The temperatures of each curve are the average values over the measurement time and the corresponding standard deviation. As observed in the results in Figure 3b, the relationship between ethanol volume fraction and the corresponding change in wavelength exhibits a non-linear trend. Nevertheless, within the volume concentration range of $0({\%}_{V/V})$ to $50{\%}_{V/V}$, all the curves can be approximated by linear fits and maintain a reliable one-to-one correspondence between the concentration and the resonance wavelength of the sensor. A linear relation $\lambda_{0}=\alpha \left(T\right)+\beta \left(T\right)\cdot C$ can be defined for each curve. Figure 3c illustrates the temperature dependence of the parameters α and β. As depicted in the figure, both the intercept and slope show a linear relationship within the tested temperature range. As a result, for a given temperature, it is possible to define the wavelength-concentration relation. To compensate for temperature variations, the sensor $S_{t}$ can be employed to measure the temperature, allowing the calculation of corresponding α and β values. By doing so, this method enables a more accurate estimation of the solution concentration compared to the results obtained when assuming a constant temperature. This is evident from the variations in the wavelength resonance of the sensor $S_{n}$ at the same concentration for different temperatures. We stress that this calibration method targets the measurement of the concentration, rather than the refractive index, of a solution in the presence of temperature changes. The compensation indeed takes into account the temperature changes of the refractive index of both the sensor material and the liquid. A separate calibration of the temperature effect on the sensor alone would allow measuring the refractive index of the liquid, but that does not directly lead to the concentration because of its temperature dependence. The proposed method is, therefore, more useful for application in the monitoring of chemical processes, where concentration is the parameter of interest.

The maximum standard deviation for the measurements in Figure 3b, where the measurements were taken every second for a duration of 8 min (480 spectra), is $\sigma_{\lambda }=12.1$ pm and corresponds to the value measured when the sensor is immersed in water and room temperature (25 °C). Considering this value of $\sigma_{\lambda }$ as the noise level of the experimental results and the calculated sensitivity for the corresponding curve (52.9 $\mathrm{pm}/{\%}_{V/V}$), the limit of detection (LoD) is estimated as LoD $=3\sigma_{\lambda }/\left(d\lambda /dC\right)=0.68{\%}_{V/V}$. Furthermore, an Allan deviation analysis was conducted on the collected data. The Allan deviation is a widely used time-domain tool for studying the noise characteristics of a sensor signal with respect to integration time or the number of data points [24]. In Figure 3d, the Allan deviation plot illustrates that the noise can be reduced by averaging multiple measurements, reaching its minimum value at a measurement time of 3 s. Based on this minimum value, a wavelength noise of $\sigma_{\lambda }=1.3$ pm and a potential limit of detection (LoD) of 0.07%_V/V_ could be achievable in principle.

### 3.2. Ethanol Concentration Measurements

**Figure 4:**
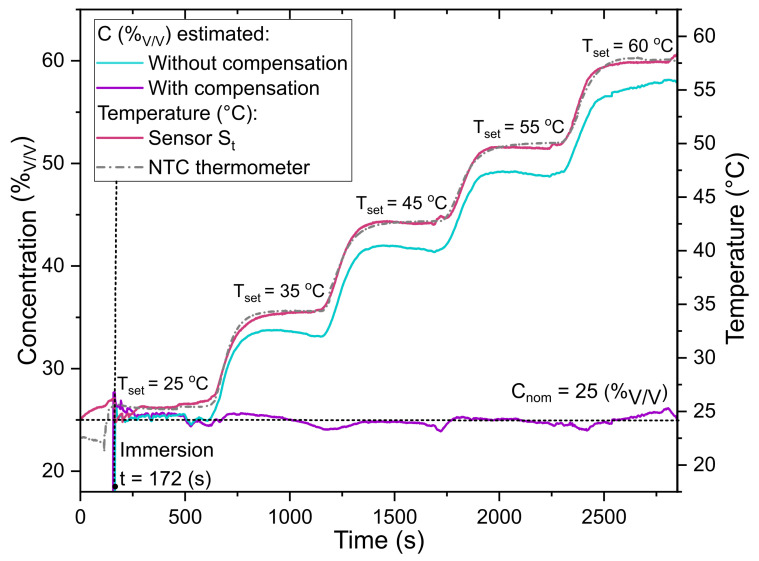
Response of both sensors and the commercial NTC thermometer during the ethanol–water mixture measurements at different temperatures. For this example, a time trace of the sensors immersed in $25\;({\%}_{V/V})$ while the set temperature is increased from 25 °C to 60 °C is shown.

To further prove that it is possible to measure the concentration of ethanol and compensate for temperature changes, the sensor probe is tested in three different volume fraction solutions (0, 25, and 50 $\left({\%}_{V/V}\right)$). To perform these experiments, the sensor was placed in an empty centrifuge tube in the temperature-controlled mixer, set at RT and 300 rpm. After approximately 2 min, 1 mL of the previously prepared ethanol-water mixture is deposited in the tube and the temperature control is fixed at 25 °C. Then, the temperature is set to 35, 45, 55 and 60 °C sequentially in intervals of 10 min for each value. The resonant wavelength is acquired every second, and the concentration and temperature values are calculated using the corresponding linear fits shown in Figure 3 and following the procedure described earlier. Figure 4 presents an illustrative time series example of temperature and concentration estimation, both with and without compensation, using sensors $S_{n}$ and $S_{t}$. In this particular example, the concentration was set to 25% while the temperature varied from 25 °C to 60 °C. For the results without compensation, the concentration estimation assumes a constant temperature of 25 °C. Additionally, the temperature acquired from the commercial NTC thermometer is included as a reference to assess the performance of the sensor $S_{t}$.

In Figure 4, it can be seen that both sensors, $S_{n}$ and $S_{t}$, behave as expected. While the temperatures obtained with $S_{t}$ closely match the temperatures measured with the commercial thermometer (see Figure 4), there is a difference between these values and the set temperature. This is because the set temperature refers to the temperature of the plate element that is underneath the tube holder, and the temperature sensors measure the temperature in the liquid. Moreover, the initial measured concentration matches with the nominal one (25 $\left({\%}_{V/V}\right)$). Nevertheless, notice that the calculated concentration without compensation increases over time when the temperature increases. This is because the effect of the temperature in $S_{n}$ is not being considered. Hence, this variation in the response of $S_{n}$ needs to be compensated in order to obtain accurate measurements of the ethanol concentration. In contrast, when considering the temperature measured by the sensor $S_{t}$ to determine the specific α and β values, and subsequently employing the corresponding linear fit of wavelength as a function of concentration ($C({\%}_{V/V})$), the temperature compensation method provides accurate estimations of the concentration (as can be seen in Figure 4). It is important to note that the estimated concentration exhibits a higher discrepancy with the nominal value when the temperature is not stable. This discrepancy can be attributed to the different time responses of both sensors during temperature transients. However, when the temperature is stable, the estimated concentration consistently matches the nominal value across the entire range of tested temperatures.

**Figure 5:**
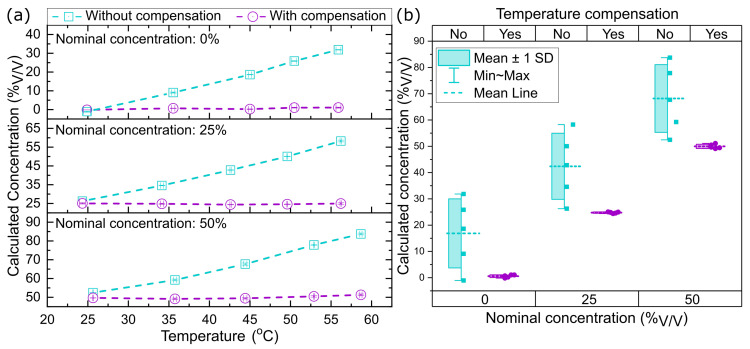
(**a**) Average of the calculated ethanol concentration of different solutions without and with compensation as a function of the temperature measured by $S_{t}$ at each plateau. (**b**) Average of the calculated ethanol concentration of different solutions without and with compensation at different temperatures. The error bars are given by the standard deviation over 300 measurements (5 min).

To perform a comparative analysis, for experiments similar to the one shown in Figure 4, the concentration of different solutions (0%, 25% and 50%) with and without temperature compensation were calculated. The results for the different concentration measurements are shown in Figure 5a. For these data, the measurements performed during the time span where the temperature is stable (5 min) are considered to obtain the average of both calculated concentrations, before and after compensation. As can be seen in Figure 5a, all the concentration values calculated before the temperature compensation show a substantial drift as the temperature increases. In contrast, the concentration values obtained after the temperature compensation are much closer to the nominal concentration. To visualize how the compensation improves the concentration measurement, Figure 5b shows the values obtained over the temperature range and the standard deviation between them. In Figure 5b, it is evident that the temperature compensation improves considerably the determination of the solution concentration regardless of the temperature. Compared with the measurements with no compensation, where the maximum error, i.e., the difference between calculated and nominal concentration, is 33.7%, the measurements after compensation show a maximum error of 1.1%. Additionally, the standard deviation of the concentration values calculated at the different temperatures ranges between 0.3% and 0.8%. The results shown in Figure 5 prove that the temperature compensation method used allows us to obtain an accurate measurement of the concentrations in a broad range of temperatures, from 25 °C to 55 °C.

## 4. Conclusions

In this paper, a highly sensitive liquid concentration sensor with temperature compensation based on two photonic crystal fiber-tip sensors was experimentally demonstrated. The two sensors are placed in a sensor probe, which allows the isolation of one of the sensors for the independent temperature measurement to compensate for the effect of this parameter in the concentration sensor measurements. Ethanol aqueous solutions with different concentrations were tested, showing a sensitivity of 52.9 $\mathrm{pm}/{\%}_{V/V}$ when the concentration ranged from 0 to $60{\%}_{V/V}$, and a limit of detection of 0.08% for an integration time of 3 s. Finally, utilizing the proposed sensing probe and temperature compensation method, the accurate determination of the concentration of the solutions at different temperatures is possible. At temperatures ranging from 25 °C to 55 °C, our results showed that the measurement of liquid concentration is at least twelve times more accurate than methods without temperature compensation.

Table 1 shows a comparison with other works that report fiber optical sensors of ethanol concentration and discuss the temperature effects. While these works include the characterization of the sensors to ethanol concentration and temperature and state the possibility of performing a compensation by considering the cross-sensitivity, they all lack the implementation of the actual compensation method. As a result, it is not possible to evaluate the performance of those temperature compensation techniques. In contrast, we experimentally demonstrate the temperature compensation of the concentration measurement and its effect on the measurement accuracy. In terms of the sensitivities to both parameters, these vary depending on the sensors. However, our sensing probe has one of the highest sensitivities to concentration without requiring a specific coating. Moreover, our device is the only one based on fiber-tip sensors, which is a major advantage in size, packaging, and single-point measurements, compared with fiber Bragg gratings (FBGs).

Moreover, based on the obtained results, our sensor probe exhibits promising potential for applications in industries such as food and beverage processing, pharmaceutical manufacturing, and ethanol fuel adulteration, where precise temperature compensation is vital for dependable concentration measurements. Looking ahead, future prospects of our research include extending the range of temperature and concentrations, assessing the performance of the sensor probe in specific applications, and exploring the feasibility of integrating this sensor probe into industrial processes.

## Figures and Tables

**Table 1:** Summary of ethanol concentration sensors that consider temperature effects.

Sensing Principle	T Compensation	Sensitivities	Ranges
Etched FBG [6]	No	3.6 pm/%_V/V_ 7.2 pm/°C	0–80%_V/V_ 3 & 20 °C
Oxide coated LPG [13]	No	760 pm/%_V/V_ 110 pm/°C	0–30%_V/V_ -
FBG (dual) [11]	No	3.6 pm/%_V/V_ 10 pm/°C	95–100%_V/V_ 35–60 °C
Tilted FBG [12]	No	3.2 pm/%_V/V_ 10 pm/°C	0–60%_V/V_ 25–50 °C
PhC sensor probe (this work)	Yes	53 pm/%_V/V_ 19.1 pm/°C	0–60%_V/V_ 25–60 °C

## Data Availability

Data underlying the results presented in this paper are not publicly available at this time but may be obtained from the authors upon reasonable request.
